# Vaginal Discharge in a Pre-pubertal Girl Posing a Diagnostic Challenge

**DOI:** 10.7759/cureus.2424

**Published:** 2018-04-04

**Authors:** Suhas Ganguli, Qing Liu, Apostolis Tsoumpariotis, Susana Rapaport, Esra Fakioglu

**Affiliations:** 1 Pediatrics, Northshore-Long Island Jewish Health System - Cohen childrens Medical Center; 2 Emergency Medicine, Flushing Hospital Medical Center; 3 Pediatric Medicine, Flushing Hospital Medical Center

**Keywords:** vaginal foreign body, vaginal discharge, vaginitis

## Abstract

Vaginal discharge in prepubescent girls is not an uncommon problem in pediatric outpatient practice. Among its various etiologies, foreign body lodgement is quite frequent in this age group. Diagnosis is sometimes forthcoming after history and physical exam, and the removal of the foreign object is followed by a prompt resolution of symptoms. However, in rare circumstances, an intravaginal foreign body may mimic other pathologies, including infections and neoplasms, as well as raising suspicion for child abuse. In such cases, diagnosis may remain unclear even after laboratory tests and imaging studies. We describe a seven-year-old girl with vaginal discharge, who needed inpatient admission, multiple imaging studies and, finally, exploration under anesthesia to confirm the diagnosis of foreign body (fecal mass) lodgement and its removal. This is a very rare case where the lodgement of an intravaginal fecal mass in a child led to such protracted symptoms requiring extensive diagnostic and therapeutic maneuvers, in the absence of any structural abnormality of the urogenital tract.

## Introduction

The lodgement of a foreign body is a common cause of vaginal discharge and vaginitis in children [1]. It accounts for about four percent of symptoms in pediatric gynaecology outpatient visits [2]. Symptoms include foul-smelling discharge, dysuria, and, less commonly, pain. The lodgement of a foreign body may result from age-appropriate exploratory behavior or sometimes, sexual abuse [1,3]. Diagnosis is usually made in most cases after a detailed history and a physical exam with/without standard imaging studies. We describe an exceptional case of vaginal discharge in a seven-year-old previously healthy girl due to the inadvertent lodgement of fecal matter in the vagina, requiring extensive diagnostic imaging studies and examination under anesthesia.

## Case presentation

A seven-year-old, previously healthy girl of normal growth and development was brought to the emergency room with persistent vaginal discharge for three months. According to the mother, the discharge was brown in color, staining the girl’s underwear, and had a fishy smell. Oral amoxicillin and topical nystatin prescribed by her pediatrician brought only minimal relief. She had also been given vinegar baths, as advised by a traditional healer. On initial physical examination by the pediatric and gynecologic teams, an intravaginal mass was suspected and she was admitted for further workup and imaging studies. There was no history of fever, rash, dysuria, urgency, hematuria, bloody vaginal discharge, bloody or loose stools, abdominal pain, or joint pain. No history of foreign object insertion was given. The patient lived with her mother, a twin brother, and another three-year-old male sibling. The mother was divorced from the father for about four years, but the father was well-involved in the children’s care. An extensive interview by health care providers and social workers yielded no suspicion for sexual abuse.

The physical examination revealed a calm and cooperative pre-pubertal girl with normal vital signs (BP: 110/60, heart rate (HR): 91 bpm, Temp: 36.8°C, respiratory rate (RR): 20, peripheral capillary oxygen saturation (SpO2): 97%) and a soft, non-tender abdomen with normal bowel sounds. On an inspection of the vagina, a brownish discharge with an offensive odor was found in scant amount. A pelvic examination by a gynecologist revealed a large, necrotic-appearing mass protruding from the introitus.

Blood tests included a normal complete blood count (white blood cell (WBC): 10,200/cu mm, Hb: 13.8 g/dl, platelets: 2,65,000/ cu mm), normal serum chemistries (glucose: 74 mg/dl, blood urea nitrogen: 11 mg/dl, creatinine: 0.3 mg/dl, sodium: 139 mEq/L, potassium: 4.2 mEq/L, chloride: 106 mEq/L). Urinalysis was unremarkable and vaginal cultures for Chlamydia trachomatis and Flexi-test for Trichomonas vaginalis were negative.

**Figure 1:**
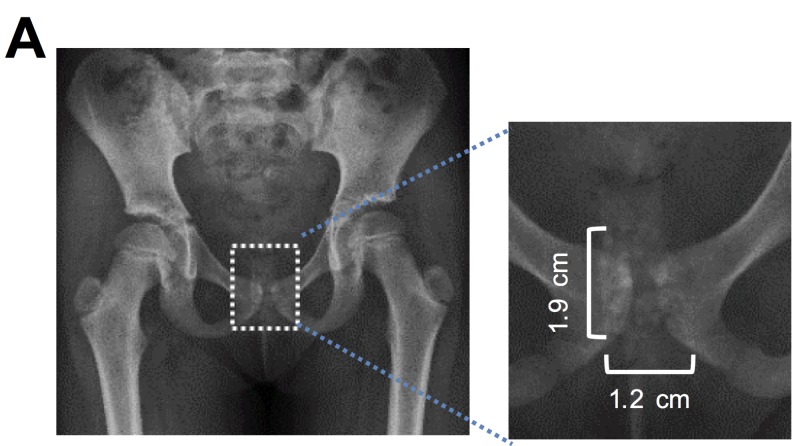
Pelvic X-ray showing a cluster of rounded calcific densities projecting over the symphysis pubis

**Figure 2:**
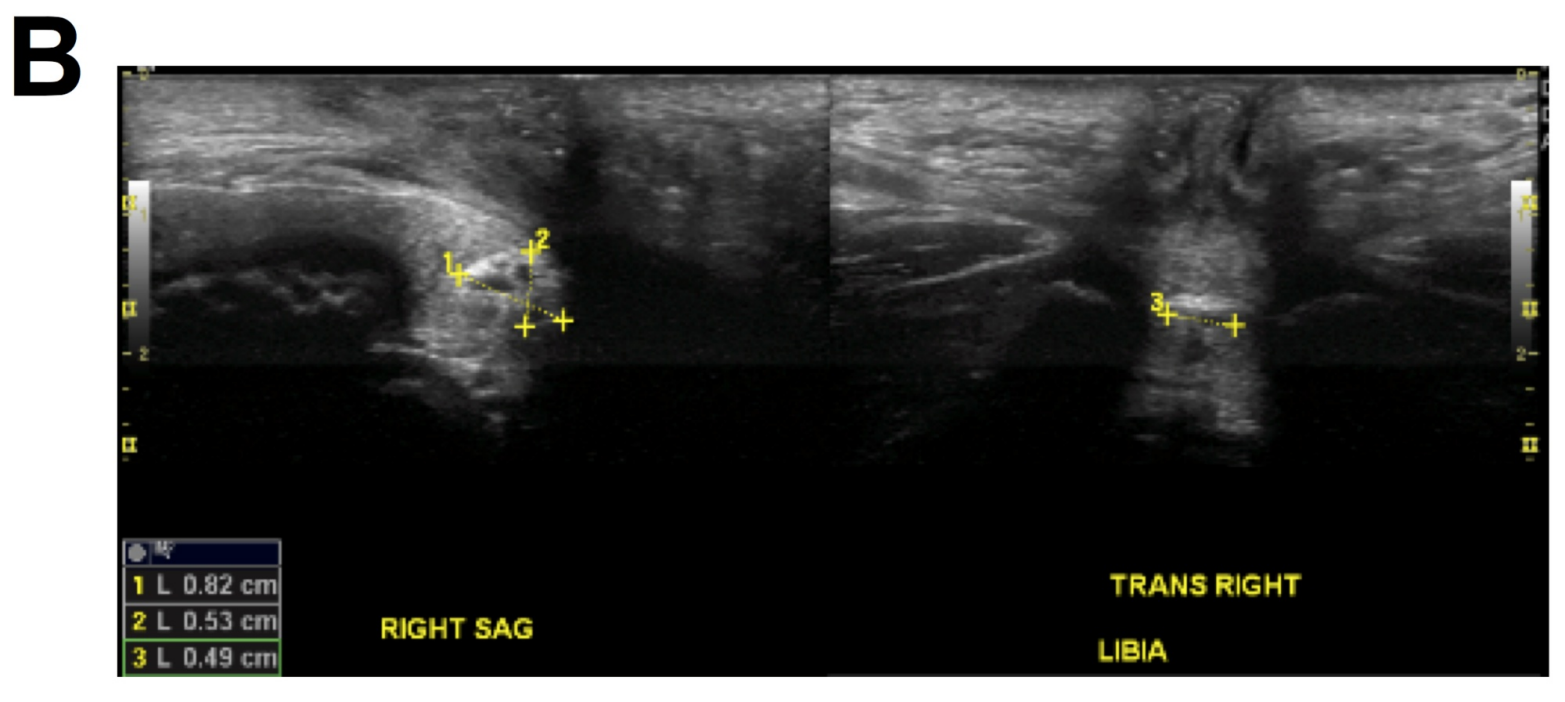
Ultrasound scan of the pelvis showed an echogenic structure measuring 0.8 cm x 0.5 cm x 0.5 cm in the region of the vagina with areas of venous flow

**Figure 3:**
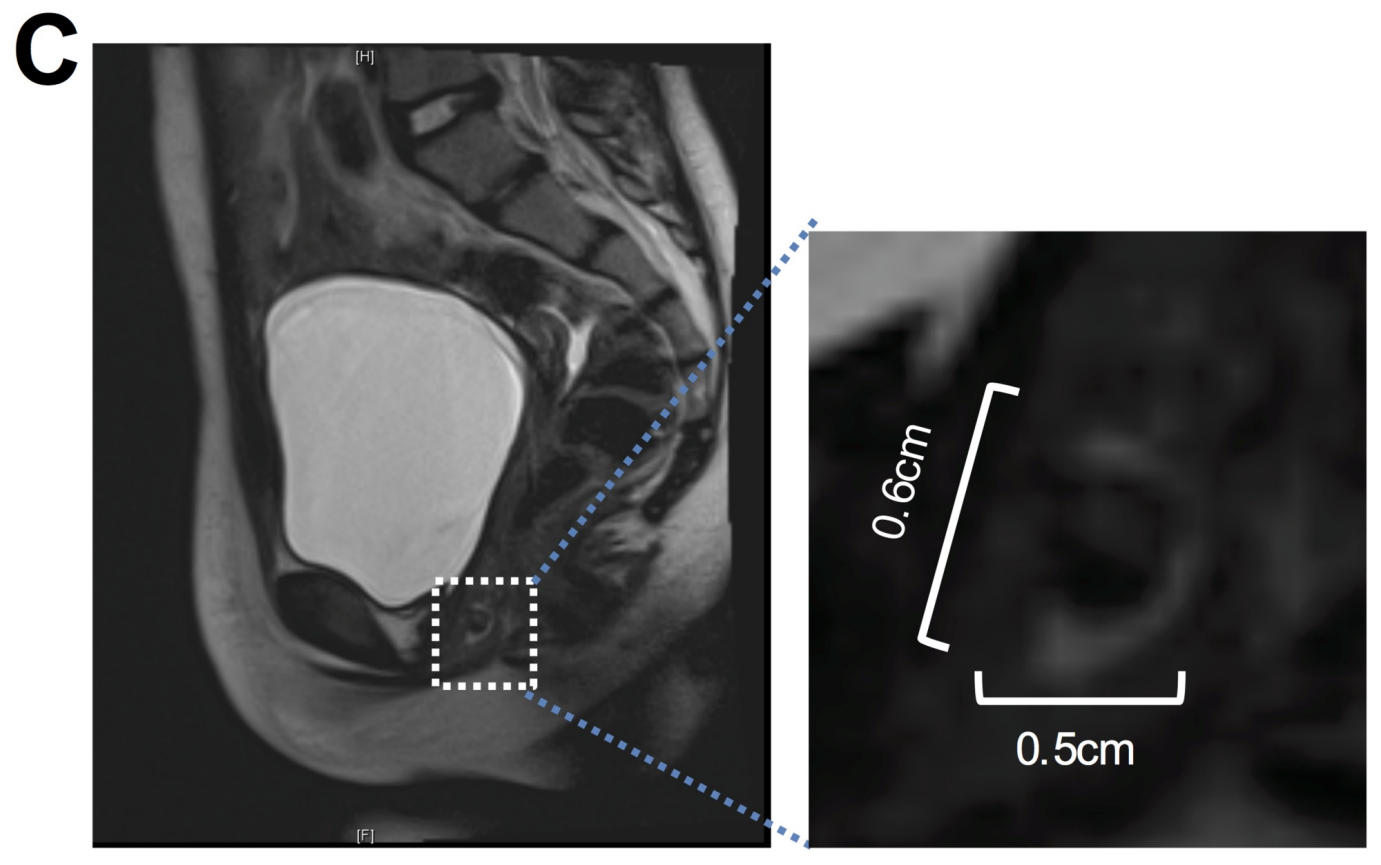
Contrast-enhanced MRI of the pelvis showing a non-enhancing filling defect within the vagina measuring 0.5 cm x 0.6 cm x 0.4 cm

An anteroposterior X-ray of the pelvis showed a cluster of rounded calcific densities projecting over the symphysis pubis, suggesting differential diagnoses of foreign object and phleboliths (Figure 1). An ultrasound scan of the pelvis showed an echogenic structure measuring 0.8 cm x 0.5 cm x 0.5 cm in the region of the vagina with areas of venous flow, without further characterization (Figure 2). Contrast-enhanced magnified resonance imaging (MRI) of the pelvis showed a small amount of fluid in the vagina and a non-enhancing filling defect within the vagina measuring 0.5 cm x 0.6 cm x 0.4 cm (Figure 3). These imaging studies raised the concerns for other possibilities, such as vaginal neoplasm and thrombus.

Since a precise diagnosis was unclear, an examination under general anesthesia was performed as the next step, which revealed a lump of dried feces in the patient’s vagina. It was removed using Debakey pick-up forceps followed by saline irrigation of the entire vagina. A rectovaginal fistula was ruled out by a negative methylene blue dye test in the operating room.

During her hospital stay, the patient received intravenous ceftriaxone and was discharged on oral cephalexin to treat for a presumed bacterial superinfection. Extensive parent and child education on personal hygiene and correct perineal cleaning techniques was provided prior to discharge.

## Discussion

Common causes of vaginal discharge (with or without a protuberant mass, as was seen in our patient) in pre-pubertal girls include vulvitis with excoriation, trauma, sexual abuse, vaginitis, vaginal foreign bodies, precocious puberty, recto-vaginal fistula, local vaginal neoplasm, sarcoma botryoides, ovarian neoplasms, such as granulosa cell tumor and germ cell tumor, or exposure to an exogenous estrogen [1]. Due to this wide range of possibilities and the urgency to rule out sexual abuse and neoplastic causes, a thorough but timely evaluation is necessary.

The prevalence of a vaginal foreign body is found to be 4% in outpatient visits in girls under 13 years of age in outpatient visits [4]. Of note, the most common intra-vaginal foreign object recovered in pediatric patients remains small wads of toilet tissue (up to 80%) [3]. Other objects described are hairpins, parts of a toy, tips of plastic markers, crayons, and gravel [1-2]. They are most commonly found in children between three and nine years of age [3] and are often due to age-appropriate curiosity and tendencies of self-exploration or introduction by playmates or siblings in up to 25% cases or, less commonly, as a result of sexual abuse [3]. The latter must be ruled out by thorough history-taking, a physical exam, diagnostic tests for sexually transmitted infections, and the involvement of an experienced social worker, as was done for our patient. Girls with developmental delays and behavioral disorders are at a higher risk of having retained vaginal foreign objects [2,5].

The classic symptoms of a vaginal foreign body are a persistent, foul-smelling discharge, often purulent due to secondary infections. They can cause local irritation and in long-standing cases, eventually embed themselves in the vaginal epithelium and bleeding or spotting may occur [2]. Often, the history of object insertion may not be forthcoming, leading to an inaccurate diagnosis and persistent symptoms for a prolonged period of time, as was pointed out in one study (n=35), where only 54% children/parents were able to recall an insertion [6]. In older children and adolescents, anxiety and embarrassment may also lead to a delay in seeking medical care.

The common complications of vaginal foreign bodies are vaginitis, frequent urinary tract infections, ulceration of vaginal walls, perforation into the abdominal cavity, formation of vesicovaginal and rectovaginal fistulae, vaginal stenosis, scarring, and, rarely, hematochezia [5-8]. It is noteworthy that a pre-existing rectovaginal fistula, in turn, can also account for the lodgement of fecal matter in the vagina. The causes of a rectovaginal fistula in children include Crohn’s disease, past surgery or radiation therapy, or Lymphogranuloma venereum [9]. In our patient, the presence of fistulae was ruled out by a negative methylene blue dye test.

A foreign body may or may not be readily visualized on physical examination. In some, knee-chest positioning may reveal small objects like a small toy or a coin. In others, vaginal swabbing under anesthesia led to the diagnosis [2]. In one case series (n=35), in about one-third of children, the foreign body was found on inspection or vaginal/rectal exam [6]. Plain radiography is limited by the fact that some of these foreign objects are not radio-opaque [2]. A pelvic computed tomogram is fraught with the risk of radiation exposure and that of adverse reaction from intravenous contrast. Ultrasound, which is safe and well-tolerated by children, can show nonspecific echogenic changes and indentation of the posterior bladder wall [10] but is confounded by inter-observer variation. Pelvic MRI is probably the most superior and accurate imaging for a vaginal mass/foreign body [3]. However, MRI is limited by a lack of availability in emergency, time, cost, and need for sedation in toddlers and younger children. In rare circumstances, even a combination of all three modalities of imaging may fail to yield an accurate diagnosis, as was seen in our patient.

Treatment is by the timely removal of the foreign object. Vaginal irrigation is often well-tolerated in cooperative children and is effective if the object is located in the distal vagina, near the introitus. There is no evidence favoring the use of modalities such as vinegar or sitz baths (as was used by our patient) or estrogen cream [5-6]. If a specific diagnosis is unclear even after imaging studies, as in our case, a vaginoscopy and/or an examination under anesthesia remains the gold standard, offering the dual advantages of diagnosis and extraction at the same time [2].

It is important during an examination under anesthesia that the physician remains vigilant for ‘red flag’ findings of possible sexual abuse: laceration of the vagina and a minor, but definite, alteration in the posterior hymenal rim.

Lastly, many believe that children with a history of lodgement of vaginal foreign bodies, especially toilet tissues, are often ‘repeat performers.’ Vaginal itching and pain sensations make them manipulate the area repeatedly. These children may benefit from the use of wipes instead of toilet tissues.

## Conclusions

Our patient is probably the first case where the inadvertent lodgement of a fecal mass, in the absence of any structural urogenital abnormality, led to such protracted symptoms, requiring an extensive workup and, finally, an examination under anesthesia for correct diagnosis and treatment. The underlying etiology of a pediatric vaginal discharge may remain elusive despite thorough history-taking, physical examination, and advanced imaging. In this age group, therefore, a high index of suspicion for foreign object (including fecal mass) lodgement must be maintained while thoroughly excluding infections, neoplasms, or sexual abuse.

## References

[1] Hillard PJA (2012). Benign Diseases of the Female Reproductive Tract. Berek and Novak’s Gynecology 15th Edition.

[2] Chinawa JM, Obu HA, Uwaezuoke SN (2013). Foreign body in vagina: an uncommon cause of vaginitis in children. Ann Med Health Sci Res.

[3] Closson FT, Lichenstein R (2013 Sep). Vaginal foreign bodies and child sexual abuse: an important consideration. West J Emerg Med.

[4] Paradise JE, Willis ED (1985). Probability of vaginal foreign body in girls with genital complaints. Am J Dis Child.

[5] Deligeoroglou E, Deliveliotou A, Laggari V, Tsimaris P, Creatsas G (2006). Vaginal foreign body in childhood: a multidisciplinary approach. J Pediatr Child Health.

[6] Stricker T, Navratil F, Sennhauser FH (2004). Vaginal foreign bodies. J Paediatr Child Health.

[7] Dahiya P, Sangwan K, Khosla A, Seth N (1999 ). Foreign body in vagina-an uncommon cause of vaginitis in children. Indian J Pediatr.

[8] Hasan M, Abdessamad MD, Greenfield M (2009). Vaginal foreign body presenting as bleeding with defecation in a child. J Pediatr Adolesc Gynecol.

[9] Al Breiky S, Al Faraidy N (2012). A Crohn’s disease rectovaginal fistula in a nine year old girl masquerading as a case of sexual abuse, and literature review. Journal of the Saudi Society of Dermatology & Dermatologic Surgery.

[10] Caspi B, Zalel Y, Katz Z, Appelman Z, Insler V (1995). The role of sonography in the detection of vaginal foreign bodies in young girls: the bladder indentation sign. Pediatr Radiol.

